# Concomitant COVID-19 and pulmonary tuberculosis: computed tomography aspects

**DOI:** 10.1590/0100-3984.2021.0070

**Published:** 2022

**Authors:** Alexandre Dias Mançano, Gláucia Zanetti, Edson Marchiori

**Affiliations:** RA Radiologia – Grupo Sabin Medicina Diagnóstica, Brasília, DF, Brazil.; Universidade Federal do Rio de Janeiro (UFRJ), Rio de Janeiro, RJ, Brazil.

**Keywords:** COVID-19, Tuberculosis, Tomography, X-ray computed, COVID-19, Tuberculose, Tomografia computadorizada

## Abstract

**Objective:**

To describe the relationship between coronavirus disease 2019 (COVID-19) and pulmonary tuberculosis during the current pandemic, as well as to describe the main computed tomography (CT) findings in patients suffering from both diseases simultaneously.

**Materials and Methods:**

This was a retrospective, cross-sectional observational study of the chest CT scans of 360 patients with COVID-19, as confirmed by RT-PCR.

**Results:**

In four (1.1%) of the patients, changes suggestive of COVID-19 and tuberculosis were observed on the initial CT scan of the chest. On chest CT scans performed for the follow-up of COVID-19, cavitary lesions with bronchogenic spread were observed in two of the four patients, whereas alterations consistent with the progression of fibrous scarring related to previous tuberculosis were observed in the two other patients. The diagnosis of tuberculosis was confirmed by the isolation of *Mycobacterium tuberculosis*.

**Conclusion:**

Albeit rare, concomitant COVID-19 and tuberculosis can be suggested on the basis of the CT aspects. Radiologists should be aware of this possibility, because initial studies indicate that mortality rates are higher in patients suffering from both diseases simultaneously.

## Abstract (pt)

**Objetivo:**

Descrever a associação entre COVID-19 e tuberculose pulmonar durante a pandemia atual e descrever os principais achados tomográficos.

**Materiais e Métodos:**

Estudo retrospectivo transversal e observacional de tomografias computadorizadas de tórax realizadas em 360 pacientes com COVID-19 confirmada por RT-PCR.

**Resultados:**

Em quatro pacientes (1,1%) foram encontradas alterações tomográficas sugestivas de associação entre COVID-19 e tuberculose. Em dois pacientes observaram-se escavações com disseminação broncogênica e em outros dois, alterações compatíveis com progressão de lesões fibrocicatriciais relacionadas a tuberculose prévia, em exames de controle para COVID-19. O diagnóstico foi confirmado pelo isolamento do *Mycobacterium tuberculosis*.

**Conclusão:**

Apesar de incomum, a associação entre COVID-19 e tuberculose pode ser sugerida com base em aspectos tomográficos, devendo os radiologistas estar atentos a esta possibilidade, pois estudos iniciais indicam aumento da mortalidade nesses pacientes.

## INTRODUCTION

After the World Health Organization declared coronavirus disease 2019 (COVID-19) to be a public health emergency, on January 30, 2020^(1)^, the medical literature was flooded with hundreds of articles on the subject. Pulmonary tuberculosis (TB) continues to be a serious public health problem^(2)^. Studies addressing various aspects of the relationship between COVID-19 and tuberculosis have recently appeared(^1^,^3^,^4^,^5^), showing that, for example, COVID-19 might lead to disease reactivation in patients previously treated for tuberculosis and that patients being treated for tuberculosis might be more susceptible to infection with severe acute respiratory syndrome coronavirus 2 (SARS-CoV-2). It has been established that some viral infections, such as measles, can also aggravate or reactivate tuberculosis, because they deplete cellular immunity^(5)^, as could also happen in COVID-19. The use of corticosteroids in the treatment of COVID-19 could also contribute to the reactivation of tuberculosis.

The initial signs and symptoms of tuberculosis overlap with those of COVID-19, making the diagnosis of the combination of the two even more challenging(^1^,^3^). Imaging methods, especially computed tomography (CT) of the chest, can play a fundamental role in this investigation, given that the morphological CT patterns have been well established for both conditions.

The aim of this study was to evaluate concomitant COVID-19 and tuberculosis. We also describe the main findings seen on chest CT scans of patients simultaneously suffering from both diseases.

## MATERIALS AND METHODS

This was a retrospective, cross-sectional observational study of chest CT scans performed in 360 consecutive patients with COVID-19, in whom SARS-CoV-2 infection was confirmed by reverse transcription-polymerase chain reaction (RT-PCR), between August 15, 2020 and April 13, 2021 at a private clinic in the administrative region of Taguatinga, within the Federal District of Brasília, Brazil. The CT scans in which there were images suggestive of tuberculosis were selected. The diagnosis was later confirmed by isolation of *Mycobacterium tuberculosis* in bronchoalveolar lavage fluid or sputum.

The examinations were performed in two 80/160-slice multidetector CT scanners (Aquilion PRIME; Canon, Tokyo, Japan), without contrast medium. The images were obtained and reconstructed in a matrix of 512 × 512 pixels, with a slice thickness of 1 mm and an interslice gap of 1 mm. For the evaluation of the lungs, windows ranging from 1,200 Hounsfield units (HU) to 2,000 HU and center levels ranging from -300 HU to -700 HU were used. For the study of the mediastinum, the windows were 350–500 HU and the center levels were 10–50 HU. Coronal and sagittal multiplanar reconstructions were also performed.

The examinations were reassessed by two thoracic radiologists, working independently, and disagreements were resolved by consensus. The criteria used in order to define the CT findings were those established in the “Illustrated Brazilian Consensus on the terminology of the descriptors and fundamental standards of chest CT”^(6)^.

Pulmonary changes related to COVID-19, as seen on CT, were defined in accordance with the criteria established by the Radiology Society of North America^(7)^ and by the Brazilian College of Radiology and Diagnostic Imaging^(8)^. The suspicion of concomitant active tuberculosis on the CT scan was based on the findings of cavitary lesions, consolidations, a tree-in-bud pattern, airspace nodules, miliary nodules, and lymph node enlargement(^9^,^10^).

## RESULTS

Among the 360 patients with a confirmed diagnosis of COVID-19, the suspicion of concomitant tuberculosis was established by CT in four (1.1%). The time from the diagnosis of COVID-19 to the suspicion of active tuberculosis on a CT scan ranged from 16 days to 9 months (one patient had a persistent cough after recovering from COVID-19 and did not seek medical attention until 9 months later).

Of the four patients evaluated, three were female and one was male. Ages ranged from 22 years to 50 years. Two of the patients reported a history of tuberculosis treatment. All four patients reported moderate dyspnea on exertion and persistent cough, which was productive in two. Only one patient reported the reappearance of fever.

**Figure 1:**
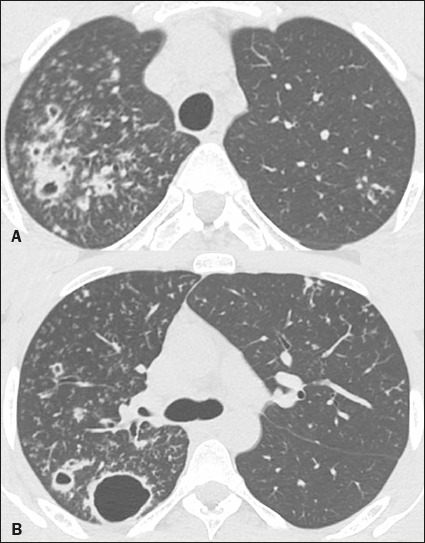
A 22-year-old man with COVID-19 and tuberculosis. Chest CT showing multiple cavitary lesions in the right upper lobe, with thickening of the bronchial walls and a tree-in-bud pattern in both upper lobes.

**Figure 2:**
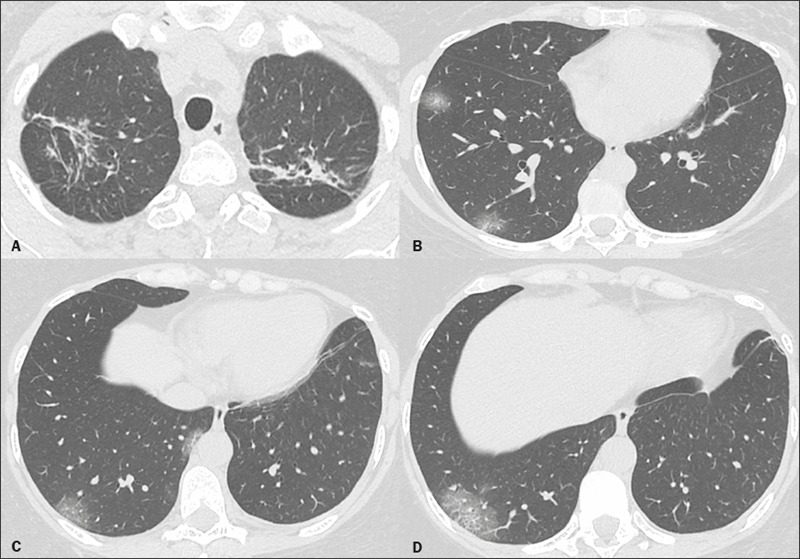
A 50-year-old woman with COVID-19 and tuberculosis. Chest CT showing fibrous scar tissue in the right upper lobe (**A**), related to the history of tuberculosis reported by the patient, and multifocal ground-glass opacities in the periphery of the lower lobes (**B–D**), related to COVID-19.

**Figure 3:**
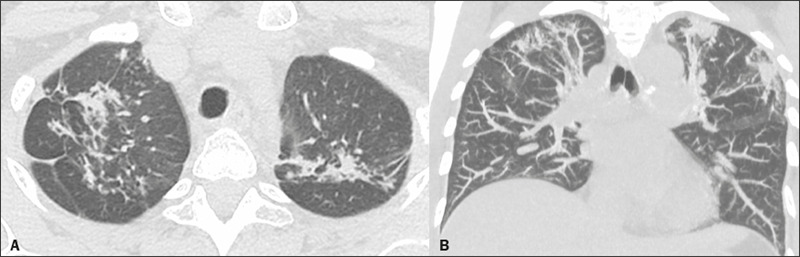
Follow-up CT scan of the patient in Figure 2, performed three months later, with axial and coronal slices (**A** and **B,** respectively), showing signs of increased reticular opacities, with architectural distortion in the upper lobes, together with new opacities and some new small irregular nodules in the right lung, indicative of tuberculosis reactivation.

In two patients, the chest CT scans showed thick-walled cavitary lesions accompanied by a tree-in-bud pattern (Figure 1), consistent with bronchogenic spread. Those patients (patients 1 and 2) did not have CT changes that could be attributed to COVID-19. In patients 3 and 4, the initial CT scan showed CT changes related to previous tuberculosis. In patient 3, that was characterized by the presence of a solid nodule with central calcification in the right upper lobe, whereas it was characterized by CT changes with aspects of fibrous scar tissue in the upper lobes in patient 4. In both, multifocal ground-glass opacities were observed in the periphery of the lower lobes (Figure 2), a finding consistent with viral pneumonia. In patient 3, a follow-up CT scan, performed three months after the initial one, showed growth of the soft tissue component of the nodule in the right upper lobe, together with the appearance of relatively small satellite nodules and a tree-in-bud pattern, which are suggestive of reactivation of tuberculosis. In patient 4, a follow-up CT, also performed approximately three months after the initial one, showed morphological changes in the lesions, with aspects of fibrous scar tissue in the upper lobes, characterized by the progression of the lesions, together with relatively small irregular nodules in the right lung (Figure 3), also suggestive of tuberculosis reactivation. The clinical picture and CT changes are summarized in Table 1.

**Table:** Summary of the clinical picture and CT findings.

Patient	Sex	Age (years)	Clinical picture at follow-up	Initial chest CT findings	Follow-up chest CT findings
1	Male	22	Dyspnea; productive cough	Not available	Cavitary lesions; tree-in-bud pattern
2	Female	30	Fever; dyspnea; productive cough	Not available	Cavitary lesions; tree-in-bud pattern
3	Female	50	Dyspnea; productive cough	Multifocal peripheral ground-glass opacities; solid nodule with central calcification	Solid nodule growth; satellite micronodules; tree-in-bud pattern
4	Female	46	Dyspnea; productive cough	Multifocal peripheral ground-glass opacities; Fibrous scar tissue in the upper lobes	Progression of fibrous scarring in the upper lobes; new, irregular nodules in the right lower lobe

In all four patients, bacteriological confirmation of tuberculosis was carried out through direct examination of bronchoalveolar lavage fluid or sputum samples. At this writing, all of the patients are being followed on an outpatient basis and are progressing well with treatment instituted.

## DISCUSSION

The COVID-19 pandemic has overburdened health care facilities throughout the world(^1^,^3^,^4^). During the pandemic, chest CT has proven to be an extremely useful tool in patients with COVID-19 pneumonia(^7^,^8^,^11^,^12^,^13^,^14^,^15^,^16^), especially for the assessment of disease progression and complications, thus alleviating some of the burden.

In COVID-19, the initial clinical picture is similar to those of many other viral and bacterial respiratory infections, including tuberculosis(^1^,^3^,^4^), although the complications that arise over the course of the disease are quite different, as is the prognosis(^3^,^4^). Tuberculosis and COVID-19 are both capable of stressing the immune system, are both transmitted through the airways, and, when suspected, can both be readily diagnosed^(1)^, rapid diagnosis being essential for the isolation of patients. In some situations, such as during the wait for laboratory results, CT, due to its speed and availability, can play a fundamental role in screening for rapid isolation, although it should be borne in mind that normal CT findings do not exclude a diagnosis of COVID-19(^7^,^8^).

Experience in the combination of COVID-19 and tuberculosis is limited(^1^,^3^,^4^). To our knowledge, the largest published study was a multicenter study, conducted in eight countries, including Brazil, and including 49 patients diagnosed with COVID-19 and tuberculosis^(4)^. Of the 49 patients evaluated in that study, 26 (53%) had tuberculosis before contracting COVID-19, 14 (28.5%) were first diagnosed with COVID-19, and nine (18.3%) were diagnosed with both diseases almost simultaneously (within a seven-day period). Of the four patients evaluated in our study, two had previously been treated for tuberculosis and showed morphological changes suggestive of tuberculosis reactivation on CT. In one of those patients, there was no report of or CT changes related to previous tuberculosis. In the other, the tuberculosis was diagnosed 16 days after the diagnosis of COVID-19 and can therefore be considered concomitant. In their study of current and former tuberculosis patients with COVID-19, Tadolini et al.^(4)^ found that 85.7% of the patients had active tuberculosis. In the present study, we found that CT showed signs of activity in all four patients, the diagnosis of tuberculosis subsequently being confirmed by bacteriological methods.

In our sample, only one patient reported fever, whereas all of the patients complained of dyspnea and persistent cough, the cough being productive in two and dry in the other two. None of the patients had previously been under suspicion of coinfection with tuberculosis. According to Guerra et al.^(3)^, the symptoms most frequently reported by patients with concomitant COVID-19 and tuberculosis are fever (in 75.0%), cough (in 62.5%), dyspnea (in 37.5%), and headache (in 37.5%). In a study of four patients with COVID-19 and tuberculosis, conducted by Tham et al.^(17)^, all of the patients presented with fever and cough, the cough being productive in two and dry in the other two—exactly as in the present study.

In their study of patients with concomitant COVID-19 and tuberculosis, Tadolini et al.^(4)^ found that, in 42.8% of the patients, the only CT manifestations were those typical of COVID-19 (multifocal ground-glass opacities, typically with a peripheral distribution), whereas the CT patterns reported to be essentially related to tuberculosis (e.g., cavitary lesions, branching micronodules, and consolidations) were seen in 46.9%. In the study conducted by Tham et al.^(17)^, CT showed irregular pulmonary opacities and cavitary lesions (in 25%), pleural effusion with atelectasis/consolida-tion (in 50%), and ground-glass opacities with interlobular septal thickening (in 25%), suggesting a combination of CT patterns that can be found in both diseases. In our sample, two patients had subpleural ground-glass opacities in the lower lobes, a pattern typical of COVID-19, together with morphological changes related to previous tuberculosis, which, on follow-up CT scans, were found to have evolved to signs of reactivation, characterized by lesion growth and the appearance of micronodules, some with a tree-in-bud pattern, implying small airway obstruction and bronchogenic spread. The other two patients had cavitary lesions, single in one and multiple in the other, with signs of bronchogenic spread, without CT changes suggestive of COVID-19. It is noteworthy that one of those two patients had been diagnosed with COVID-19 nine months prior, whereas the other had been diagnosed just 16 days prior. No pleural effusion or mediastinal lymph node enlargement was observed in any of the patients in our sample.

According to Tham et al.^(17)^, the reported mortality among patients with concomitant COVID-19 and tuberculosis ranges from 11.6% to 33.3%, being 12.3% overall in the study conducted by Tadolini et al.^(4)^ in which it was found to be higher in patients over 60 years of age and in those with at least one comorbidity. In both of those studies, mortality was much higher in the patients with concomitant COVID-19 and tuberculosis than in those with COVID-19 alone. All of the patients in our sample were being treated as outpatients and had favorable outcomes.

Our study has some limitations, many of which are related to the retrospective, observational design and the fact that all of the patients were treated at the same facility. In conclusion, we showed that tuberculosis was present in 1.1% of patients with COVID-19. Albeit uncommon, the combination of the two diseases increases the mortality rate, and early diagnosis is essential for institution of the appropriate therapy. Because CT plays an important role in raising suspicion of the diagnosis, radiologists should be aware of the possibility that COVID-19 and tuberculosis can coexist. Given the advancing pandemic and the increasingly frequent use of corticosteroids in patients with COVID-19, further studies are needed in order to assess the real impact of this combination.
